# Reliability and validity of the Chinese version of the retrospective family unpredictability scale

**DOI:** 10.3389/fpsyg.2025.1539693

**Published:** 2025-07-31

**Authors:** Wei Qi, Chumeng Xing, Luojin Ding, Jingli Wang, Dong Guo, Yiye Xu

**Affiliations:** ^1^Department of Psychology, Zhejiang Sci-Tech University, Hangzhou, Zhejiang, China; ^2^School of Marxism, Beijing Institute of Fashion Technology, Beijing, China; ^3^Mental Health Education and Counseling Center, Shanghai University of Medicine and Health Sciences, Shanghai, China; ^4^Institute of Sociology, Shanghai Academy of Social Sciences, Shanghai, China

**Keywords:** retrospective family unpredictability, Chinese version, revision, reliability, validity

## Abstract

**Objective:**

This study aimed to revise the Chinese version of the Retrospective Family Unpredictability Scale (Retro-FUS-C) and evaluate its psychometric properties.

**Methods:**

Two independent samples were collected through convenience sampling. Sample 1 (*N* = 277) was used for item analysis, parallel analysis, and exploratory factor analysis. Sample 2 (*N* = 612) was used to conduct confirmatory factor analysis, assess internal consistency, and examine criterion-related validity.

**Results:**

The revised Retro-FUS-C retained 18 items and identified a three-factor structure: parental discipline, maternal nurturance, and paternal nurturance. Confirmatory factor analysis supported the three-factor model with good model fit, and measurement invariance across gender was supported. Internal consistency was satisfactory (Cronbach's α = 0.76-−0.90; McDonald's ω = 0.85−0.95). Significant correlations with external measures supported criterion-related validity.

**Conclusion:**

The Retro-FUS-C provides a reliable and culturally appropriate tool for assessing retrospective family unpredictability among Chinese young adults, with potential utility in identifying individuals at risk for later psychological difficulties and informing prevention and intervention efforts.

## 1 Introduction

The unpredictability in the early life environment significantly impacts an individual's physical and psychological development. According to life history theory, individuals assess the harshness (danger or poverty) and unpredictability of their early environments to forecast future survival or reproductive conditions, and adjust their psychological and behavioral strategies accordingly to enhance fitness (Belsky et al., 2012; Doom et al., 2016; Ellis et al., 2009; Maner et al., 2023). Family unpredictability represents a critical aspect of early environmental unpredictability. It is defined as inconsistent family behavioral patterns and regulatory systems, in which parents are unable or unwilling to consistently fulfill caregiving responsibilities (e.g., providing emotional support and comfort) or maintain predictable disciplinary practices (e.g., punishment without misbehavior) (Ross and Hill, 2000). Disparities in family unpredictability can lead to differences in cognitive and behavioral patterns. Individuals exposed to high family unpredictability often develop the belief that the world is chaotic and unreliable, leading them to allocate resources to the present rather than the future, resulting in increased risk-taking behaviors (Griskevicius et al., 2013). Such behavioral adaptations, while potentially functional in unstable environments, are also associated with negative physical and mental health outcomes (Qi et al., 2024; Ross et al., 2016). Therefore, accurately assessing family unpredictability is essential for understanding individual psychological functioning, behavioral tendencies, and health risks.

Existing research primarily measures early life environmental unpredictability using retrospective self-report questionnaires (Mittal et al., 2015; Young et al., 2018). For example, Mittal et al. developed three items to assess the level of chaos in early life environments. These brief scales have been widely applied and have shown good reliability (Chen and Kruger, 2017; Feng et al., 2023), yet they have not undergone systematic validation. Moreover, they primarily assess general environmental chaos, rather than the specific and multifaceted nature of family unpredictability. Ross and Hill (2000) developed the Family Unpredictability Scale (FUS) to assess the degree of inconsistency in family behavioral patterns and regulatory systems. It consists of 22 items and comprises four factors: discipline, nurturance, meals, and money. The discipline factor (7 items) reflects inconsistency in establishing and maintaining rules; the nurturance factor (7 items) reflects parental inconsistency in offering comfort, protection, care, and emotional support during stressful situations; the meals factor (5 items) reflects uncertain meal times and companions; the money factor (3 items) reflects economic instability. The scale demonstrated good reliability and validity, and has been well validated in North American populations (Ross and Hill, 2004).

The FUS is a parent self-report scale that measures parents' perceptions of family unpredictability and is commonly used to examine its effects on child development. To more accurately assess the impact of early family unpredictability on adult outcomes, it is essential for adults to report their childhood experiences retrospectively. To this end, Ross and McDuff (2008) developed the Retrospective Family Unpredictability Scale (Retro-FUS), which asks participants to recall their perceptions of family unpredictability before age 18. Initial validation of the scale was conducted with a sample of 416 college students (aged 18–39, *M* = 19.86, *SD* = 2.39). Confirmatory factor analysis supported a six-factor model comprising 28 items, including meals (5 items), money (3 items), mom discipline (4 items), dad discipline (4 items), mom nurturance (6 items), and dad nurturance (6 items), with correlated errors specified for similar mom and dad items related to nurturance and discipline. The internal consistency of the subscales ranges from 0.71 to 0.84. Retro-FUS is a widely used and well-validated measure of family unpredictability in Western contexts, with strong evidence supporting its reliability and validity (Kolak et al., 2018; Luo et al., 2022; Ross et al., 2016; Ross and Wynne, 2010).

Due to potential cultural differences, the concept of family unpredictability may not generalize across cultures. Alarcão and Gaspar (2007) examined the FUS in a Portuguese sample of 514 mothers of children and adolescents aged 2 to 18. Although the revised structure was consistent with the original FUS, the internal consistency of the meal dimension was low (α = 0.55), raising concerns about cross-cultural applicability. To our knowledge, no studies have revised the retrospective version of the scale (Retro-FUS). However, Howat-Rodrigues et al. (2012) developed a Brazilian instrument based on the Retro-FUS and other local measures, titled *Imprevisibilidade Familiar na Infância* (EIFI), a 27-item scale rated on a 5-point Likert scale. Exploratory factor analysis yielded four factors: care/support, money, meals, and discipline, though discipline items cross-loaded onto the care dimension. In 2014, further validation supported a three-factor model comprising nurturance, money, and meals, which explained 53.37% of the variance (Howat-Rodrigues and Tokumaru, 2014). These findings underscore the importance of considering cultural context when measuring family unpredictability, regardless of whether it is assessed retrospectively or in the present, and suggest that directly applying Western-developed scales in China may pose risks in terms of item relevance and structural validity.

Currently, no studies in China have developed or revised tools for measuring family unpredictability. Despite the growing interest in understanding how family unpredictability influences psychological and behavioral outcomes, many researchers have directly adopted Western-developed instruments without formal validation. For example, Luo et al. (2022) employed the Environmental Unpredictability Questionnaire (Mittal et al., 2015) and the Family Unpredictability Scale (FUS; Ross and Hill, 2000) to examine the relationship between early environmental unpredictability and adult overeating. However, only six FUS items were used, excluding those related to meals and money, possibly reflecting cultural differences. These practices raise concerns about cultural applicability and psychometric soundness in China.

Significant differences in dietary practices, financial behavior, and family structures make it difficult to apply Western-developed questionnaires to the Chinese context directly. In terms of dietary norms, Chinese culture places greater emphasis on dining etiquette and shared experiences. In financial behavior, Chinese families tend to prioritize saving, while Western cultures are more inclined toward anticipatory consumption. For instance, China has one of the highest household savings rates globally (Song et al., 2021), yet its credit participation remains relatively low, with only 28% to 32% of households using credit between 2007 and 2016, compared to 74% to 78% in the United States (Survey and Research Center for China Household Finance and Ant Group Research Institute, 2019). Regarding family structures, China's long-standing family planning policies have resulted in smaller household sizes. According to the recent Chinese General Social Survey (2021), 78.9% of families have only one or two children. In contrast, Western families typically have more children, which require parents to distribute their attention and resources more broadly. Such differences in family size and structure inevitably shape child-rearing practices and parental behavior. Given these cultural differences, it is necessary to revise the Chinese version of the Retro-FUS and evaluate its psychometric properties to ensure its suitability for the Chinese context.

This study aims to evaluate the psychometric properties of the Retro-FUS-C through a two-phase design. First, the original Retro-FUS was translated into Chinese, and exploratory factor analysis (EFA) was conducted on a sample of Chinese adults to examine item characteristics and underlying factor structure. Second, confirmatory factor analysis and reliability testing were conducted with an independent sample to confirm the factor structure and internal consistency. We also tested the measurement invariance for gender. Based on prior research linking childhood environmental unpredictability and harshness to adult attachment styles and psychological outcomes (Ellis et al., 2009; Ross et al., 2016; Ross and McDuff, 2008), we further examined correlations between the Retro-FUS-C, childhood socioeconomic status, neighborhood danger, adult attachment dimensions, and depressive symptoms.

## 2 Method

### 2.1 Participants

This study was conducted in accordance with the ethical principles of the Declaration of Helsinki and was approved by Zhejiang Sci-Tech University Psychology Research Ethics Committee (No. 202211P001). Prior to participation, all participants provided informed consent online after being fully informed of the study's purpose, procedures, and potential risks.

Participants were recruited via convenience sampling from participant pools at two universities in Zhejiang and Shanghai, using Questionnaire Star (https://www.wjx.cn), a widely used online survey platform in mainland China. These pools included participants from various family backgrounds, geographic regions, and socioeconomic levels across China. Although university-based, this sampling approach ensured heterogeneity and supports preliminary generalizability.

Two independent samples were collected. Sample 1 included 277 participants (40.1% male; age range = 18–28 years, *M* = 20.90, *SD* = 2.23) and was used for item analysis, parallel analysis, and exploratory factor analysis. Sample 2 included 612 participants (57.7% male; age range = 18–47 years, *M* = 20.56, *SD* = 2.82) and was used for confirmatory factor analysis, reliability analysis, and criterion-related validity testing. Data were collected at ~2-week intervals, with exclusion criteria including age under 18 and invalid responses (e.g., patterned answers). Participants also reported demographic information, including gender, family residence (urban/rural), only-child status, and monthly per capita family income. Descriptive statistics are presented in Table 1.

**Table 1:** Demographic characteristics of participants (*N* = 889).

**Demographic characteristic**	**Code**	**Sample A (*****N*** = **277)**		**Sample B (*****N*** = **612)**	
		***N***	**%**	***N***	**%**
**Gender**					
Male	1	111	40.07	353	57.68
Female	2	166	59.93	259	42.32
**Family residence location**					
Urban	1	171	61.73	394	64.38
Rural	2	106	38.27	218	35.62
**Only-child status**					
One child	1	130	46.93	309	50.49
With siblings	2	147	53.07	303	49.51
**Per capita family income monthly (RMB)**					
¥0–2,500	1	12	4.33	16	2.61
¥2,501–7,500	2	64	23.10	149	24.34
¥7,501–12,500	3	82	29.60	218	35.62
¥12,501–17,500	4	55	19.86	130	21.24
¥17,501–22,500	5	26	9.39	54	8.82
¥22,500	6	38	13.72	45	7.35

### 2.2 Measures

#### 2.2.1 Retrospective family unpredictability scale

The Retrospective Family Unpredictability Scale (Retro-FUS) was developed by Ross and McDuff (2008) to assess perceived unpredictability in the family environment before the age of 18. The original scale consists of 28 items rated on a 5-point Likert scale ranging from 1 (not at all) to 5 (extremely), covering six dimensions: dad nurturance, mom nurturance, dad discipline, mom discipline, meals, and money. Items 1, 2, 3, 4, 5, 8, 9, 12, 13, 14, 17, 18, 19, 22, and 23 are reverse-scored. After reverse scoring, higher total scores reflect greater perceived family unpredictability.

After being authorized by the original developers, the scale was translated into Chinese by a Ph.D. in psychology and then back-translated into English by bilingual researchers with backgrounds in psychology. Five rounds of expert group discussions were conducted to ensure semantic and conceptual equivalence, reaching consensus. Additionally, 10 college students completed the Retro-FUS-C to verify its clarity and readability.

#### 2.2.2 Childhood socioeconomic status scale

The Childhood Socioeconomic Status Scale (CSES), developed by Griskevicius et al. (2013), consists of three items rated on a 7-point Likert scale (1 = strongly disagree, 7 = strongly agree). Total scores range from 3 to 21, with higher scores indicating higher perceived childhood socioeconomic status. The Cronbach's α in the current study was 0.88.

#### 2.2.3 Adult attachment scale

This study employed the Chinese version of the Adult Attachment Scale (AAS; Wu and Fang, 2004), which is based on the original scale developed by Collins and Read (1990). The AAS includes three subscales: Close, Depend, and Anxiety, each rated on a 5-point Likert scale ranging from 1 (strongly disagree) to 5 (strongly agree). Higher Close scores reflect greater comfort with intimacy; higher Depend scores reflect a stronger tendency to rely on others when needed; and higher Anxiety scores reflect higher levels of worry about being rejected or unloved. In the current study, Cronbach's α coefficients for the three subscales were as follows: Close (α = 0.74), Depend (α = 0.60), and Anxiety (α = 0.89).

#### 2.2.4 Center for epidemiologic studies depression scale

The Center for Epidemiologic Studies Depression Scale (CES-D; Radloff, 1977) is a 20-item self-report measure developed by the National Institutes of Health to assess depressive symptoms in the general population. Participants rated the frequency of symptoms experienced during the past week on a 4-point scale ranging from 1 (rarely or none of the time, < 1 day) to 4 (most or all of the time, 5–7 days). Higher total scores indicate greater levels of depressive symptoms (Ross et al., 2022). The Cronbach's α of this study was 0.94.

#### 2.2.5 Neighborhood danger

A single item assessing neighborhood danger was included in the survey: “Which best describes the neighborhood where you lived the longest?” Responses were rated on a 5-point Likert-type scale, with 1 indicating “very unsafe with very high crime” and 5 indicating “very safe with very low crime” (Ross and McDuff, 2008).

### 2.3 Data analysis

Data were analyzed using SPSS 25.0, AMOS 24.0, and R 4.2.1 (R Core Team, 2022). Participants were divided into two groups. Sample 1 was used for item analysis, followed by parallel analysis (PA) to determine the number of common factors, and then exploratory factor analysis (EFA) using SPSS 25.0 (Messick, 2000). Sample 2 was used for confirmatory factor analysis (CFA) to examine structural validity using AMOS 24.0. Model fit was evaluated using standard indices. The chi-square statistic (χ^2^) assesses overall model fit but is sensitive to sample size; therefore, χ^2^/df < 5 was used as a criterion for acceptable fit. Comparative fit index and Tucker-Lewis index (CFI; TLI; ≥ 0.90 acceptable, ≥ 0.95 good) compare the proposed model to a baseline model, with higher values indicating better relative fit. Root mean square error of approximation (RMSEA; ≤ 0.06 good, ≤ 0.08 acceptable) estimates error per degree of freedom, and standardized root mean square residual (SRMR; ≤ 0.08) reflects the average residual between observed and predicted correlations (Hu and Bentler, 1999). Measurement invariance across gender was tested using multi-group confirmatory factor analysis (MG-CFA) at the configural, metric, scalar, and strict levels. Invariance was considered acceptable if the change in model fit met the recommended thresholds (ΔCFI < 0.01; ΔRMSEA < 0.015; Chen, 2007). Internal consistency reliability was assessed using Cronbach's alpha (α), calculated in SPSS, and McDonald's omega (ω), along with its 95% confidence interval, computed in R 4.2.1. Values of α or ω ≥ 0.70 were considered acceptable, and ≥ 0.80 indicated good reliability (DeVon et al., 2007; Hayes and Coutts, 2020). Criterion-related validity was assessed through correlation analysis using SPSS 25.0.

## 3 Result

### 3.1 Item analysis

First, item analysis was conducted on the original Retro-FUS. Items 1, 2, 3, 4, 5, 8, 9, 12, 13, 14, 17, 18, 19, and 23 were reverse-coded. We then calculated the corrected item-total correlation (CITC), which reflects the correlation between each item and the total score of the remaining items in the same subscale (Chen et al., 2015). After four iterations, seven items with low CITC (*r* < 0.3) were deleted (Chen et al., 2015), i.e., item 6 (0.27), item 24 (0.21), item 27 (0.26), item 7 (0.28), item 17 (0.29), item 3 (0.24), item 28 (*r* = 0.30, rounded from 0.298). The remaining 21 items in the scale all had acceptable CITC (0.31 to 0.56). We also calculated the item-total correlation (ITC), defined as the correlation between each item and the overall total score (Hao and Hong, 2014), which ranged from 0.39 to 0.56 (*p* < 0.01). To further test item discrimination, the top and bottom 27% of scorers were compared using independent-samples *t*-tests, and all items showed significant group differences (*p* < 0.05).

### 3.2 Exploratory factor analysis

Before conducting exploratory factor analysis (EFA), we assessed data suitability. The KMO value was 0.71 (>0.60; Kaiser, 1974) and Bartlett's test was significant (χ^2^ = 2,849.36, *p* < 0.001; Bartlett, 1954), supporting factorability. Parallel analysis supported a three-factor structure. EFA was conducted using principal component extraction with varimax rotation. Item 12 was removed due to low factor loading (< 0.40; Hair et al., 2010), and items 8 and 9 were removed because they loaded on the same factor, leading to conceptual overlap between maternal and paternal nurturance. The final three-factor model explained 53.26% of the total variance, with all retained items loading strongly (>0.50) on their respective factors without significant cross-loadings. The three factors were: (1) Parental discipline: reflecting inconsistency in establishing and maintaining rules by both parents. (2) Maternal nurturance: reflecting inconsistency in the mother's responsiveness to the child's needs. (3) Paternal nurturance: reflecting inconsistency in the father's responsiveness to the child's needs (see Table 2).

**Table 2:** Results of exploratory factor analysis for the Retro-FUS-C (*N* = 277).

**Items**	**Factor load**		
	**1**	**2**	**3**
20. Whether or not my mom disciplined me when I acted up depended on her mood at the time. 20. 在我调皮时妈妈是否管教我取决于她当时的心情。	0.76		
21. Whether or not my dad disciplined me when I acted up depended on his mood at the time. 21. 在我调皮时爸爸是否管教我取决于他当时的心情。	0.75		
10. How my mom acted in a specific situation depended on her mood. 10. 我妈妈在特定情况下的行为取决于她的情绪。	0.72		
11. How my dad acted in a specific situation depended on his mood. 11. 我爸爸在特定情况下的行为取决于他的情绪。	0.67		
16. Sometimes my dad yelled at me without thinking about what he was saying. 16. 有时我爸爸不假思索地对我大喊大叫。	0.67		
15. Sometimes my mom yelled at me without thinking about what she was saying. 15. 有时我妈妈不假思索地对我大喊大叫。	0.66		
25. How my mom would act from one situation to another was unpredictable. 25. 我无法预料我妈妈在不同情境里的行为。	0.61		
26. How my mom would act from one situation to another was unpredictable. 26. 我无法预料我爸爸在不同情境里的行为。	0.59		
13. When I got my feelings hurt, I went to my mom for comfort. 13. 我伤心时会找妈妈寻求安慰。		0.79	
22. When something was bothering me, I told my mom about it. 22. 遇到困扰时我会告诉妈妈。		0.76	
18. My mom let me know on a regular basis that I was important to her. 18. 我妈妈总是让我知道我对她很重要。		0.71	
4. When I had an injury, I went to my mom for first aid. 4. 我受伤时会第一时间找妈妈处理伤口。		0.67	
1. My mom spent time with each individual child each day. 1. 我妈妈每天都会花时间在每个孩子身上。		0.60	
23. When something was bothering me, I told my dad about it. 23. 遇到困扰时我会告诉爸爸。			0.86
14. When I got my feelings hurt, I went to my dad for comfort. 14. 我伤心时会找爸爸寻求安慰。			0.80
4. When I had an injury, I went to my dad for first aid. 4. 我受伤时会第一时间找爸爸处理伤口。			0.76
2. My dad spent time with each individual child each day. 2. 我爸爸每天都会花时间在每个孩子身上。			0.62
19. My dad let me know on a regular basis that I was important to him. 19. 我爸爸总是让我知道我对他很重要。			0.53

Factor 1, Parents discipline; Factor 2, Maternal nurturance; Factor 3, Paternal nurturance.

### 3.3 Confirmatory factor analysis

To evaluate the structural validity of the scale, a series of confirmatory factor analyses (CFAs) were conducted on Sample 2. Model fit indices for all tested models are presented in Table 3. We first tested a six-factor model with correlated errors (Model 1), based on Ross and McDuff's (2008) original structure and including all items. The model showed a poor fit (χ^2^*/df* = 5.90, RMSEA = 0.09, CFI = 0.84, TLI = 0.82, SRMR = 0.15), and several items in the Money and Food dimensions had factor loadings below 0.5, suggesting that these items may not be suitable for assessing family unpredictability in the Chinese context. Next, we tested a four-factor model based on Ross and McDuff's (2008) structure using the reduced item set from Sample 1 (Model 2), and the same model with correlated errors added (Model 3). Both models demonstrated poor fit, indicating that separating paternal and maternal discipline as distinct dimensions may not be appropriate.

**Table 3:** Results of confirmatory factor analysis for the Retro-FUS-C.

**Model**	**Study/Model source**	**Items**	**Factors**	**Corr. Error**	***χ^2^***	***df***	***χ^2^/df***	**TLI**	**CFI**	**SRMR**	**RMSEA**
1	Ross and McDuff (2008)	28	Six	Yes	1,960.57	333	5.90	0.82	0.84	0.15	0.09
2	Ross and McDuff (2008)	18	Four	No	2,499.70	166	14.76	0.65	0.69	0.14	0.15
3	Ross and McDuff (2008)	18	Four	Yes	853.33	156	5.47	0.89	0.91	0.14	0.09
4	Our study	18	Three	No	1,545.26	132	11.71	0.76	0.79	0.06	0.13
**5**	**Our study**	**18**	**Three**	**Yes**	**406.75**	**123**	**3.31**	**0.95**	**0.96**	**0.04**	**0.06**
6	Our study (male)	18	Three	Yes	233.42	123	1.90	0.97	0.97	0.04	0.05
7	Our study (female)	18	Three	Yes	356.29	123	2.90	0.90	0.92	0.05	0.09

Corr. Error, correlated errors for similar maternal and paternal items about nurturance and discipline; χ^2^, chi-square statistic; df, degrees of freedom; TLI, Tucker–Lewis Index; CFI, Comparative Fit Index; SRMR, Standardized Root Mean Square Residual; RMSEA, Root Mean Square Error of Approximation; Model 5 (bolded) represents the best-fitting model based on multiple fit indices.

**Figure 1:**
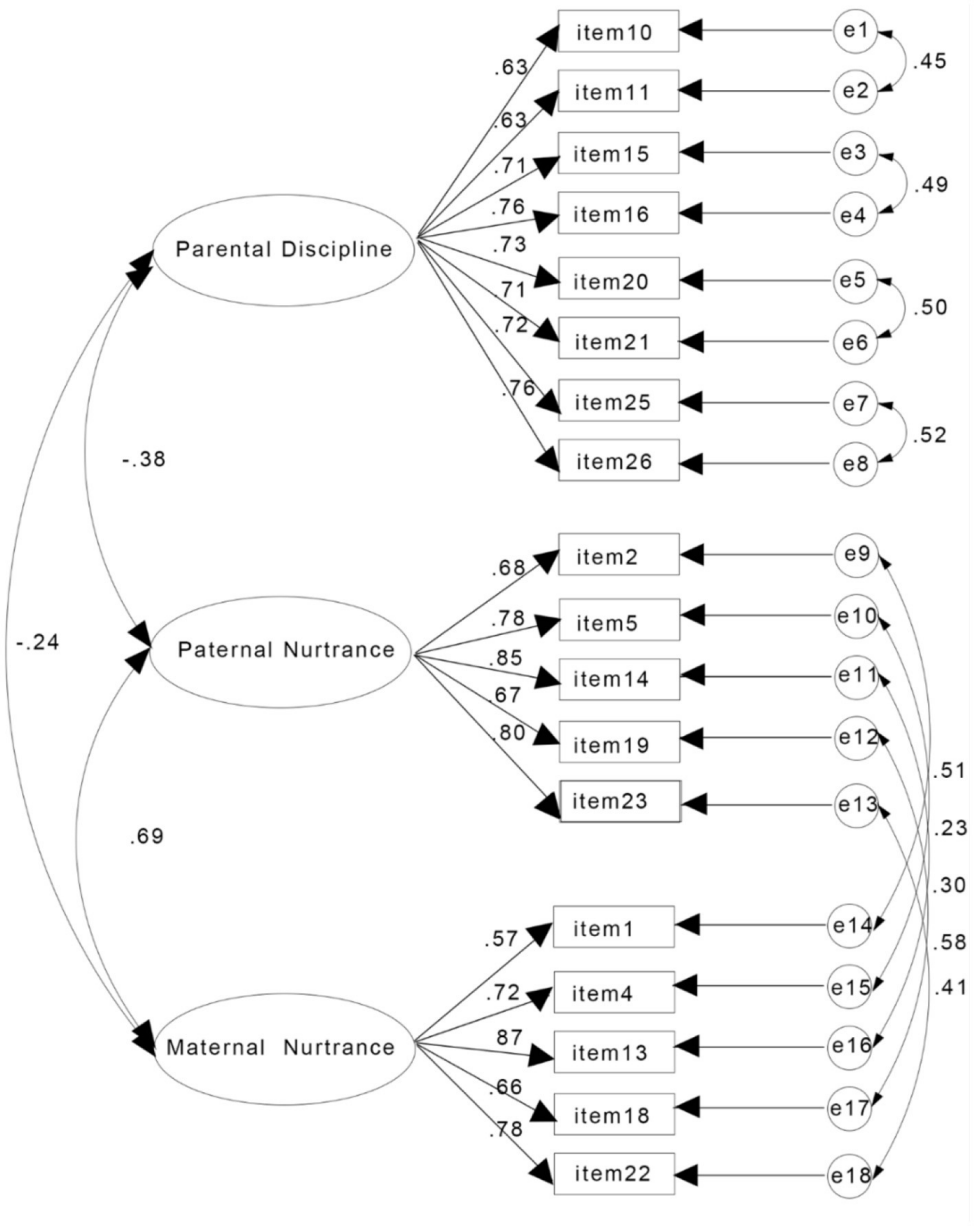
Final confirmatory factor analysis model of the Retro-FUS-C.

We then tested a three-factor model derived from the exploratory factor analysis (Model 4), which showed an unsatisfactory fit. To improve model fit, we also incorporated the correlated error terms between similar maternal and paternal items. No additional residual correlations were introduced beyond those theoretically justified in the original structure. This revised three-factor model (Model 5) showed a good fit (χ^2^*/df* = 3.31, RMSEA = 0.06, CFI = 0.96, TLI = 0.95, SRMR = 0.04), exceeding that of both the original and alternative structures. The factor loadings of this model were all >0.5 (see Figure 1). Given its strong fit and consistently high factor loadings, Model 5 was selected as the final measurement model for subsequent analyses.

### 3.4 Measurement invariance across gender

To assess model stability across gender, Model 5 was tested separately in male (Model 6) and female (Model 7) subsamples. Both models demonstrated acceptable fit (see Table 3). Although the RMSEA for the female group was 0.09, slightly exceeding the commonly recommended threshold of 0.08, it falls within the “mediocre fit” range (0.08–0.10) proposed by Browne and Cudeck (1993) and was considered acceptable. To further test measurement equivalence across gender, a series of multi-group confirmatory factor analyses (MG-CFA) were conducted to test configural, metric, scalar, and strict invariance. As shown in Table 4, the configural model demonstrated good fit (χ^2^ = 548.63, *d*f = 242, CFI = 0.955, RMSEA = 0.046), indicating that the overall factor structure was comparable across male and female groups. The metric invariance model, which constrained factor loadings to be equal, showed no change in CFI (ΔCFI = 0.000) and a slight improvement in RMSEA (ΔRMSEA = 0.002), supporting metric invariance. Scalar invariance was also supported, with ΔCFI = 0.008 and ΔRMSEA = −0.003, both within the commonly accepted thresholds (ΔCFI < 0.01, ΔRMSEA < 0.015; Chen, 2007). Finally, strict invariance was established, as the changes in fit indices remained minimal (ΔCFI = 0.002, ΔRMSEA = 0.001). These results indicate that the Retro-FUS-C demonstrated full measurement invariance across gender at all tested levels.

**Table 4:** Measurement invariance of the Retro-FUS-C across gender.

**Fitness**	***χ^2^***	***df***	**CFI**	**RMSEA**	**ΔCFI**	**ΔRMSEA**
Configural	548.634	242	0.955	0.046	–	–
Metric	561.935	257	0.955	0.044	0.000	0.002
Scalar	618.980	263	0.947	0.047	0.008	−0.003
Strict	662.496	292	0.945	0.046	0.002	0.001

χ^2^, chi-square statistic; df, degrees of freedom; CFI, Comparative Fit Index; RMSEA, Root Mean Square Error of Approximation; ΔCFI, change in CFI between nested models; ΔRMSEA, change in RMSEA between nested models.

### 3.5 Reliability analysis

The internal consistency of the Retro-FUS-C was supported by both Cronbach's α and McDonald's ω coefficients (see Table 5). For the total scale, Cronbach's α was 0.76 and McDonald's ω was 0.95 [95%CI (0.94, 0.96)], indicating high latent reliability despite a moderate alpha. For the subscales, Cronbach's α ranged from 0.85 to 0.90, and ω ranged from 0.85 to 0.89, all within acceptable to excellent levels. These results suggest that the total scale and subscales of the Retro-FUS-C demonstrate strong internal consistency.

**Table 5:** Internal consistency reliability of the Retro-FUS-C and its subscales.

**Scale**	**Cronbach's α**	**McDonald's ω**	**95% CI for ω**
Total retro-FUS-C	0.76	0.95	[0.94, 0.96]
Maternal nurturance	0.90	0.85	[0.77, 0.90]
Paternal nurturance	0.87	0.87	[0.83, 0.91]
Parental discipline	0.85	0.89	[0.87, 0.91]

### 3.6 Criterion-related validity

To evaluate the criterion-related validity of the Retro-FUS-C, Pearson correlations were conducted between its total score and subscale scores and a set of theoretically relevant external variables, including childhood socioeconomic status (CSES), perceived neighborhood danger, adult attachment styles (Close, Depend, Anxiety), and depressive symptoms (CES-D). As shown in Table 6, the total score of the Retro-FUS-C was negatively correlated with CSES and neighborhood danger, indicating that individuals who perceived higher unpredictability in their families tended to come from less advantaged and riskier environments. It was also negatively associated with Close and Depend, and positively associated with Anxiety. In addition, the total score was positively associated with depressive symptoms. Similar patterns were observed across the three subscales, particularly with parental discipline showing the strongest associations with anxiety attachment (*r* = 0.60) and depressive symptoms (*r* = 0.56). These findings reflect a theoretically consistent pattern of associations, mostly in the moderate range, supporting the Retro-FUS-C's criterion-related validity.

**Table 6:** Correlations between the Retro-FUS-C and criterion variables (*N* = 612).

**Variables**	**1**	**2**	**3**	**4**	**5**	**6**	**7**	**8**	**9**	**10**
1. Retro- FUS-C	–									
2. Maternal nurturance	0.63^***^	–								
3. Paternal Nurturance	0.55^***^	0.66^***^	–							
4. Parental discipline	0.54^***^	−0.20^***^	−0.32^***^	–						
5. CSES	−0.37^***^	−0.46^***^	−0.44^***^	0.07	–					
6. Neighbor danger	−0.14^***^	−0.13^**^	−0.07	−0.06	0.22^***^	–				
7. Close	−0.48^***^	−0.23^***^	−0.13^**^	−0.39^***^	0.29^***^	0.15^***^	–			
8. Depend	−0.48^***^	−0.19^***^	−0.07	−0.44^***^	0.21^***^	0.12^**^	0.67^***^	–		
9. Anxiety	0.46^***^	0.06	−0.10^*^	0.60^***^	−0.07	−0.06	−0.54^***^	−0.65^***^	–	
10. CES-D	0.44^***^	0.07	−0.08^*^	0.56^***^	−0.15^***^	−0.17^***^	−0.53^***^	−0.59^***^	0.62^***^	–

CSES, Childhood Socioeconomic Status Scale; CES-D, Center for Epidemiologic Studies Depression Scale.

^*^p < 0.05, ^**^p < 0.01, ^***^p < 0.001.

## 4 Discussion

This study revised and validated the Chinese version of the Retrospective Family Unpredictability Scale (Retro-FUS-C) for use in the Chinese cultural context. With permission from the original author, we translated the scale and conducted item analysis, exploratory factor analysis (EFA), and confirmatory factor analysis (CFA). EFA suggested a three-factor structure, and CFA supported the modified model with good fit indices. Measurement invariance across gender was also established, indicating that the scale functions equivalently for male and female respondents. In addition, the Retro-FUS-C demonstrated acceptable internal consistency and criterion-related validity, supporting its reliability and utility in Chinese samples.

The three-factor structure of the Retro-FUS-C includes maternal nurturance, paternal nurturance, and parental discipline, consistent with the original scale's dimensions of nurturance and discipline proposed by Ross and McDuff (2008). However, there was a minor difference: the dimensions of paternal and maternal discipline were combined. This may reflect a more uniform perception of parental discipline in the Chinese context. Unlike the more differentiated parenting styles often observed in Western cultures, Chinese parents are strongly influenced by Confucian values that emphasize obedience, harmony, and filial piety. Within this cultural framework, discipline is frequently expressed through a “training” approach (Chao, 1994), which integrates behavioral control with emotional closeness and high parental involvement. Consequently, mothers and fathers in China often adopt similar disciplinary strategies. Supporting this, Luo et al. (2021) found through cluster analysis that over two-thirds of Chinese families exhibited high consistency between maternal and paternal parenting styles. In contrast, nurturance is more closely tied to culturally gendered parenting roles. Meta-analytic findings suggest that mothers are generally viewed as the primary caregivers, providing more emotional and day-to-day support, whereas fathers are more often associated with instrumental roles such as financial provision (Dou et al., 2020). Thus, while discipline reflects a shared parental identity, nurturance tends to retain distinct maternal and paternal characteristics.

Additionally, items related to meals and money were excluded due to both cultural and psychometric considerations. In terms of dietary practices, Western cultures tend to adopt square tables or buffet-style dining, reflecting cultural traits of independence and individuality, whereas Chinese culture emphasizes communal dining and family cohesion, typically around round tables (Ma, 2015; Ye et al., 2021). Rooted in Confucian values, shared family meals are viewed as rituals of filial piety and are maintained even under unpredictable circumstances (Christensen, 2017; Tian et al., 2018). As a result, unpredictability in family meals may not be applicable in the Chinese context. Similarly, the money items primarily assessed short-term financial fluctuations (e.g., “Some months we had plenty of money to spend, other months we were quite poor”), which may not be culturally appropriate in the Chinese context. In Western societies, where pre-consumption and credit-based spending is common, temporary income instability tends to have a stronger psychological impact. In contrast, Chinese families typically emphasize saving and frugality over unplanned consumption (Xiao and Fan, 2002; Ye et al., 2021), reflecting longstanding cultural values. This is further evidenced by data from the 2016 China Household Finance Survey (CHFS), which showed that only 17% of Chinese households owned credit cards (Gan et al., 2019), suggesting limited reliance on personal credit. Therefore, items referencing uncertainty about covering monthly expenses, such as “My parents were never sure how we would pay our bills from month to month,” may not resonate with most Chinese respondents. Moreover, financial uncertainty is often mitigated by intergenerational transfers and extended family support (Meng et al., 2023; Silverstein and Zhang, 2020), as well as relatively stable employment in government and state-owned sectors (Li et al., 2024; Wang and Xie, 2015). These structural and cultural factors reduce the psychological salience of financial volatility. Psychometrically, these items showed low CITC and/or weak factor loadings, supporting their exclusion on both cultural and empirical grounds.

Although the money dimension (assessing financial fluctuations) was excluded, our analyses confirmed that lower CSES remained significantly associated with higher family unpredictability. This result aligns with life history theory's distinction between harshness (stable adversity, e.g., chronic low CSES and neighborhood risk) and unpredictability (variable conditions, e.g., unstable nurturance) as related but distinct developmental antecedents (Belsky et al., 1991, 2012; Ellis et al., 2009; Griskevicius et al., 2013; Simpson et al., 2012). This suggests that chronic economic hardship, resource scarcity, and environmental threats may undermine the stability of parenting behaviors and emotional support (Belsky et al., 1991), ultimately shaping an individual's developmental trajectories.

According to classical attachment theory (Bowlby, 1969), consistent and predictable caregiving is essential for the development of a secure attachment system. In contrast, when caregivers respond inconsistently, children may become uncertain about their availability, which fosters insecure attachment patterns such as anxiety and avoidance (Ainsworth et al., 1978). These insecure patterns can later contribute to difficulties in forming and maintaining close relationships in adulthood (Fraley and Shaver, 2000; Hazan and Shaver, 1987). Contemporary attachment theorists highlight that early relational experiences shape internal working models, which guide expectations and behaviors in adult relationships (Mikulincer and Shaver, 2007). In this context, the unpredictability (such as inconsistent nurturance and discipline, measured by Retro-FUS-C) may interfere with the development of stable internal working models of relationships (Barbaro and Shackelford, 2016; Belsky et al., 1991; French et al., 2020). Our findings are consistent with this framework, as higher scores of Retro-FUS-C were associated with weaker emotional bonds in adulthood (Close and Depend) and increased abandonment anxiety. These findings underscore the utility of the revised Retro-FUS-C in retrospectively assessing early family unpredictability and identifying individuals at risk for relational difficulties, thereby informing targeted interventions in close relationships.

Although most studies using the Retro-FUS have focused on non-clinical samples, emerging evidence suggests that early-life unpredictability contributes to psychological distress, particularly depression (Kolak et al., 2018; Ross et al., 2016; Wang et al., 2024). For example, Ross et al. (2016) found that greater exposure to family unpredictability predicted higher levels of depressive symptoms in adulthood. In our study, the significant association between higher unpredictability and elevated depressive symptoms underscores the potential clinical relevance of the Retro-FUS-C. Future research should investigate whether this scale can reliably predict psychiatric conditions such as mood and anxiety disorders in clinical populations.

In summary, this study demonstrates that the Retro-FUS-C is a reliable and culturally appropriate tool for assessing retrospective family unpredictability among Chinese young adults. By supporting key predictions of life history theory and attachment theory, it contributes to the understanding of early environmental influences in Chinese contexts. The scale also shows potential value in identifying individuals at heightened risk for later psychological difficulties, which may inform future prevention and intervention efforts. However, this study has certain limitations. Although participants were recruited from university participant pools, they came from diverse geographic regions, family backgrounds, and socioeconomic levels across China. This sampling approach, consistent with that used in the development of the original scale, supports initial generalizability. Nevertheless, the revised scale is currently more applicable to Chinese young adults, and its relevance to middle-aged or older populations remains unclear. Future research should consider age-specific revisions if necessary. Second, with the increasing popularity of consumer credit tools in China, such as Ant Credit Pay and installment-based purchasing, the experience and perception of financial unpredictability among younger generations may continue to change. As a result, the financial items in the Retro-FUS may require further revision over time to maintain contextual relevance. Future studies should examine how evolving economic behaviors affect the validity of these items and adapt the scale as new cohorts enter adulthood.

## 5 Conclusion

This study comprehensively evaluated the psychometric properties of the Chinese version of the Retrospective Family Unpredictability Scale (Retro-FUS-C). The results indicate that the Retro-FUS-C demonstrates good reliability and validity and is a culturally appropriate tool for assessing family unpredictability among Chinese young adults. It offers researchers and healthcare professionals in China a scientifically sound instrument to investigate early environmental influences and their psychological consequences. Further research is encouraged to explore the scale's applicability across different age groups and populations.

## Data Availability

The datasets presented in this study can be found in online repositories. The names of the repository/repositories and accession number(s) can be found below: https://osf.io/g6yvh/?view_only=d16bf2a505e94bbc8be89ff477cd7570.
